# The potential of a self-assessment tool to identify healthcare professionals’ strengths and areas in need of professional development to aid effective facilitation of group-based, person-centered diabetes education

**DOI:** 10.1186/s12909-017-1003-3

**Published:** 2017-09-18

**Authors:** Vibeke Stenov, Gitte Wind, Timothy Skinner, Susanne Reventlow, Nana Folmann Hempler

**Affiliations:** 1 Department of Nursing, Metropolitan University College, Tagensvej 86, DK-2200 Copenhagen N, Denmark; 2 School of Psychological and Clinical Sciences, Charles Darwin University, Darwin, NT 0909 Australia; 3 The Research Unit and Department of General Practice, University of Copenhagen, Øster Farimagsgade 5, DK-1014 Copenhagen K, Denmark; 4 Health Promotion, Steno Diabetes Center Copenhagen, Niels Steensens Vej 2, DK-2820 Gentofte, Denmark

**Keywords:** Person-centered methods, Group-based patient education, Diabetes, Ethnographic fieldwork, Qualitative methods, Professional skills, Educator behavior, Communication skills

## Abstract

**Background:**

Healthcare professionals’ person-centered communication skills are pivotal for successful group-based diabetes education. However, healthcare professionals are often insufficiently equipped to facilitate person-centeredness and many have never received post-graduate training. Currently, assessing professionals’ skills in conducting group-based, person-centered diabetes education primarily focus on experts measuring and coding skills on various scales. However, learner-centered approaches such as adequate self-reflective tools have been shown to emphasize professional autonomy and promote engagement. The aim of this study was to explore the potential of a self-assessment tool to identify healthcare professionals’ strengths and areas in need of professional development to aid effective facilitation of group-based, person-centered diabetes education.

**Methods:**

The study entails of two components: 1) Field observations of five different educational settings including 49 persons with diabetes and 13 healthcare professionals, followed by interviews with 5 healthcare professionals and 28 persons with type 2 diabetes. 2) One professional development workshop involving 14 healthcare professionals. Healthcare professionals were asked to assess their person-centered communication skills using a self-assessment tool based on challenges and skills related to four educator roles: Embracer, Facilitator, Translator, and Initiator. Data were analyzed by hermeneutic analysis. Theories derived from theoretical model ‘The Health Education Juggler’ and techniques from ‘Motivational Interviewing in Groups’ were used as a framework to analyze data. Subsequently, the analysis from the field notes and interview transcript were compared with healthcare professionals’ self-assessments of strengths and areas in need to effectively facilitate group-based, person-centered diabetes education.

**Results:**

Healthcare professionals self-assessed the Translator and the Embracer to be the two most skilled roles whereas the Facilitator and the Initiator were identified to be the most challenged roles. Self-assessments corresponded to observations of professional skills in educational programs and were confirmed in the interviews.

**Conclusion:**

Healthcare professionals self-assessed the same professional skills as observed in practice. Thus, a tool to self-assess professional skills in facilitating group-based diabetes education seems to be useful as a starting point to promote self-reflections and identification of healthcare professionals’ strengths and areas of need of professional development.

**Electronic supplementary material:**

The online version of this article (10.1186/s12909-017-1003-3) contains supplementary material, which is available to authorized users.

## Background

Patient education is a critical element of care for all people with diabetes [1]. In particular, a person-centered approach in diabetes education has been shown to successfully support long-term behavioral changes and enhance quality of life among people with type 2 diabetes (T2DM) [2, 3]. Evidence suggests that healthcare professionals’ (HCPs) person-centered communication skills are pivotal for successful self-management in individuals with T2DM [4].

Most person-centered approaches have been developed for use by HCPs conducting individual consultations, although group-based diabetes education is a commonly used self-management approach because it brings people with T2DM together to share experiences and is optimally cost-effective [5–9]. Person-centered approaches are critical components of successful group programs. However, incorporating these approaches into practice requires a wide range of professional skills. HCPs must both adopt a more facilitative approach to addressing group members’ experiences, needs, and concerns and be skilled in managing group dialog to ensure a supportive and collaborative group atmosphere [10]. In this study, we define professional skills as the ability to perform high-quality group-based, person-centered diabetes education in practice.

It is often difficult for HCPs to support group-based, person-centered diabetes programs due to a lack of ongoing professional development and supervision [4, 11, 12]. Recent results from the second Diabetes, Attitudes, Wishes and Needs (DAWN2) study revealed that HCPs were inadequately equipped to provide diabetes education, and many had never received postgraduate training [4]. Such training is a key element in developing person-centered professional skills that enable HCPs to undertake new roles and successfully facilitate group-based diabetes education [13–15].

Currently, HCPs’ skills at delivering person-centered education are evaluated by experts who rate professional communication skills using expert-designed coding scales [16–20]. An expert-dominated approach to assessment can foster tension and create conflict; HCPs may interpret it as judgmental and confrontational and respond in guarded, defensive, and superficial ways, limiting their acquisition of new skills and behaviors [21–24]. Assessments in which experts dominate and provide recommendations and advice on specific actions are morally directed and can impair, rather than improve, person-centered professional skills [25, 26].

To promote professional autonomy and engagement, it is essential to support HCPs in identifying their needs and challenges related to facilitating group-based, person- centered diabetes self-management education [23, 27]. Translating group-based, person-centered approaches into professional skills calls for the development of learner-centered approaches including nonjudgmental methods such as robust self-reflection tools [24, 28]. These approaches enable HCPs to reflect on their skills and encourage self-assessment and self-problem solving first as they seek to improve their professional skills [22].

The aim of this study was to explore the potential of a self-assessment tool to identify HCPs’ professional strengths and areas in need of professional development to aid effective facilitation of group-based, person- centered diabetes education.

## Methods

The qualitative study was conducted between March 2015 and October 2016. It entailed two components: 1) field observations of HCPs from five educational programs in the Greater Copenhagen area of Denmark, followed by interviews with program participants with T2DM and HCPs; and 2) using insights gained from the field observations and interviews, a professional development workshop for HCPs was conducted focusing on self-assessments of skills required to deliver group-based, person-centered diabetes education. To explore the potential of a self-assessment tool to identify HCPs’ strengths and areas in need of professional development to aid effective facilitation of group-based, person-centered diabetes education, we compared field notes and interview transcripts with HCPs’ self-assessments.

**Fig. 1:**
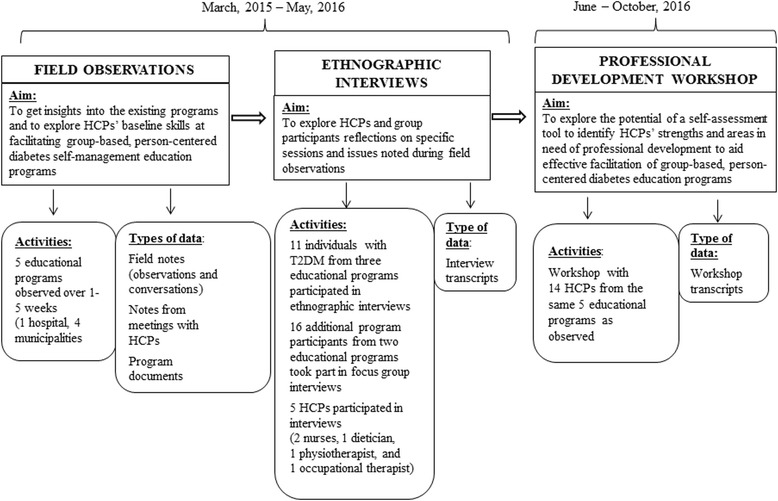
Flow chart of data collection methods

We used a tool to self-assess professional skills based on the theoretical model ‘The Health Education Juggler’ [10] and techniques from Motivational Interviewing (MI) in Groups [23]to delineate the essential elements of facilitating high-quality group-based, person-centered diabetes education (Fig. 1).

### Data collection

#### Field observations

The aim was to explore HCPs’ baseline skills at facilitating group-based, person-centered diabetes education. The HCPs had different resources, techniques, and facilities e.g. access to training class and educational tools such as a conversation map, a dialog tool box etc. Throughout the programs, the first author both participated in social activities such as casual conversations and exercises, and observed, while maintaining the analytical and intellectual distance needed to interpret social settings and recording field notes [29, 30]. Thus, the first author’s participation can be characterized as moderate [31].

Fieldwork findings informed the following interviews and focus groups exploring two issues in more depth: HCPs and program participants’ reflections on specific sessions and issues noted during observations. Thus, the field observations and interviews gained insights into HCPs needs and challenges in existing practice. The knowledge obtained from the ethnographic study and interviews informed the following professional development workshop. The workshop were planned to meet the needs and challenges of HCPs.

By initially investigating challenges in practice and subsequently involving the HCPs in professional development, the workshop aimed at bridging the gap between research and practice. 

#### Professional development workshop

The professional development workshop reported in this article is part of a larger study consisting of three workshops in total focusing on developing professional skills to facilitate group-based, person-centered diabetes education. However, this particular study presents the findings of the first workshop where the aim was to stimulate HCPs’ self-reflection about their professional skills by identifying their strengths and areas in need. Skills were identified with the tool to self-assess professional skills (Table 1). In the second workshop, the aim was to develop methods supporting HCPs in facilitating group-based, person-centered diabetes education, whereas the last workshop aimed at evaluating and redesigning group-based, person-centered methods after being tested in practice.

**Table 1:** Overview of activities in workshop 1

Method	Process
Icebreaking and brainstorming process	A written and verbal exercise to stimulate initial reflections on how HCPs currently facilitate group-based, person-centered diabetes education (plenary discussion)
Self-assessing professional skills	Brief presentation of the Four Health Education Roles (plenary) Self-assessing the most skilled and most challenged Health Education Roles. HCPs were asked to merely mark one skilled and one challenged role (Fig. 2) (individual)
Small group discussions followed by plenary discussions	To identify how HCPs applied the roles. They were asked to discuss in pairs the roles they had chosen and why. Furthermore, to explain how they managed their strengths and challenges in practice
Cases, discussions in small groups followed by plenary discussions	In the perspective of the Four Health Education Roles the HCPs were asked to identify common challenges observed by the researcher in practice
Questionnaire (Additional file 1)	Level of experience and postgraduate training Current use of group-based, person-centered methods Assess HCPs’ readiness/willingness to incorporate group-based, person-centered methods (individual)

**Fig. 2:**
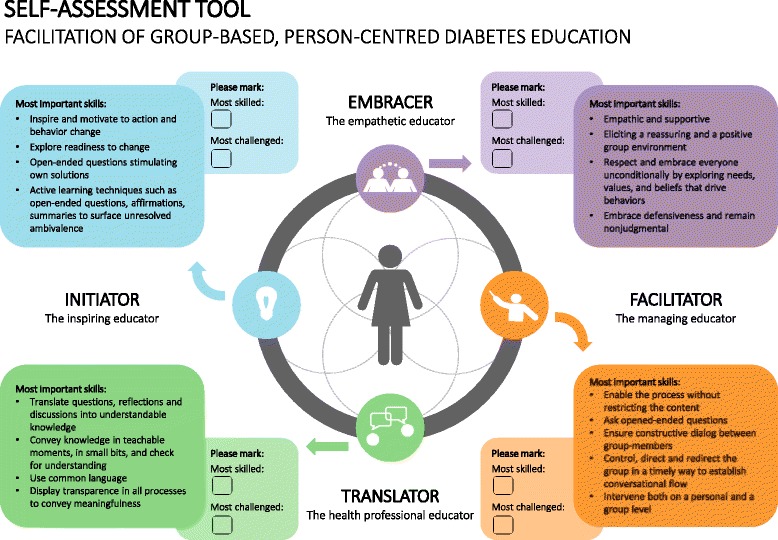
Tool to self-assess professional skills in facilitating group-based, person-centered diabetes education

Using the self-assessment tool, HCPs focused on challenges and skills related to four roles that are equally necessary to facilitate group-based, person-centered diabetes education [10]: Embracer, Facilitator, Translator, and Initiator. Key components were transferred into the tool to self-assess professional skills using practical techniques from Motivational Interviewing (MI) in Groups (Fig. 2).

The focus was to develop a learner-centered approach supporting self-reflection and enabling HCPs to identify their strengths and areas in need to effectively facilitate group-based, person-centered diabetes education programs.

All HCPs had different professional backgrounds and level of postgraduate training. Although all HCPs had experience with delivering group-based diabetes education, they had not received formal supervision. However, all HCPs answered a questionnaire where they considered themselves to be highly ready to incorporate new strategies for facilitating group-based, person-centered diabetes education (T﻿able 2).

**Table 2:** Characteristics of healthcare professionals participating in the professional development workshop

Background	Male	Female
Registered ﻿nurse		4
Physiotherapist		4
Dietician	1	4
Occupational therapist	1	
Level of postgraduate training		
1 year of education at university level		1
2 weeks educational course at diploma level	2	4
2–3 days patient education course		4
No training in patient education		3

### Data analysis

Data were analyzed hermeneutically to acknowledge the interconnected nature of analysis and theory generation in the interpretation of data [30]. All data were iteratively analyzed and interpreted using the Health Education Juggler Model and techniques from MI in Groups as the theoretical and interpretative frame. Initial analysis of the field notes focused on HCPs’ teaching and conversational approaches, as well as the dialog and interaction between HCPs and participants with T2DM. The subsequent analysis of semi-structured and focus group interviews emphasized participants’ perceptions and reflections on HCPs’ teaching and conversational approach, and dialog and interaction within groups. Field notes and quotes from interviews were compared to structure the data within the framework of the four Health Education Juggler roles. Finally, workshop data were analyzed to compare the findings from observations with HCPs’ self-assessments. All findings were categorized into two themes representing skills about which HCPs felt most skilled and those that were most challenged, which we then divided into four subthemes related to the four Health Education Juggler roles. As a last analytical step, central concepts from MI in Groups was adapted to provide a more in-depth analysis and interpretation of the subthemes. There are several different techniques to use from MI in Groups, generally aspects such as: promoting unconditional regards; rolling with resistance; asking, listening, and informing; supporting self-efficacy for change; and using a four phases group approach (engagement, exploring perspectives, broadening perspectives, moving into action) [23].

To ensure transparency and trustworthiness of the analysis we made a varied sample by observing multiple settings and combined data from various sources. Thus, the researchers’ interpretations of field notes informed the interviews where findings were confirmed and compared with participants in the interviews including patients and HCPs with different background, years of experience, and level of continuing education. Moreover, to strengthen the analytical generalizability results were interpreted using The Health Education Model and techniques from MI in Groups [32]. Finally, quotes were used to illustrate the presented interpretations from field notes as well as interviews. Nevertheless, the hermeneutic perspective concludes that no study can provide findings that are universally transferable because it cannot be interpreted independently from its context. Yet, to enhance the transparence of the study we have included a thorough description of the research process [33–35].

## Results

### Professional skills HCPs felt skilled about

During workshops, HCPs self-assessed professional skills within the Translator and Embracer roles to be the two most skilled roles. The observations showed equally that HCPs were particular skilled in the Translator role and were highly up to date with disease-specific knowledge and used interactive learning techniques frequently. Moreover, they found it fairly easy to have an empathic attitude to ensure a supportive and collaborative group atmosphere. HCPs self-assessments corresponded to observations of most skilled professional roles during educational programs and were confirmed in interviews.

#### Translating diabetes knowledge successfully

Observations of HCPs’ professional skills during educational programs revealed that they easily adopted the Translator role. In particular, they were up-to-date about advanced theoretical and disease-specific knowledge and disseminated detailed information to the group. Theoretical knowledge was presented through didactic education focused on communicating disease-specific knowledge, and HCPs also successfully translated diabetes-specific knowledge in ways that were more readily accessible to participants, using a variety of techniques to promote interactive learning. Participants highly valued group activities such as learning to buy healthy groceries, cooking diabetes-friendly food, and physical exercise. Several participants noted that they found interactive and experience-based learning techniques very meaningful in terms of translating diabetes knowledge into their own life. After a group physical exercise activity, one program participant stated:

*The half hour brisk walk indeed decreased the blood sugar significantly. That was really an eye-opener. (Interview participant 2)*

Some HCPs were also very conscious about the importance of using common language instead of technical and medical terms when they conveyed diabetes-specific knowledge. In one program, an HCP appeared keenly aware of the methods for delivering information. The Diabetes Conversation Map™ [36] was used as a learning tool to actively involve and engage participants in the educational process by emphasizing conversations about diabetes-specific topics. The map was used to help the group to more easily retain information and understand different aspects of T2DM through visuals and metaphors. The HCP asked questions concerning the pictures on the map. Although there was not enough time to cover the whole map, it was important for the HCP to investigate what the group knew by asking questions and then filling in knowledge gaps using manageable amounts of information, instead of lecturing extensively. The following excerpt from the field notes describes how the educator translated diabetes-specific knowledge:

*A visual map entitled ‘How Diabetes Works’ was used. Series of images on the map described the physical condition of T2DM. The map illustrated a factory producing keys as a metaphor for the pancreas producing insulin. Furthermore, the map illustrated a cell with T2DM where the keys were unable to unlock the transportation of sugar into the cells due to dysfunctional keyholes – designed as a dry and withered apple tree encircled by a locked fence with blocked keyholes. The HCP asked questions like, “What does the factory illustrate?”, “What happens in the cells?”, and “What is the difference between type 1 and type 2 diabetes?” (May 2015)*

#### Embracing the group and creating a reassuring environment

Many HCPs were particularly skilled in fulfilling the Embracer role. They greeted the group with a relaxed and kind attitude, which served as an ice breaker and stimulated a positive and safe group climate. Program participants appreciated the ability of HCPs to acknowledge and normalize the challenges of diabetes management through a nonjudgmental attitude. As one program participant stated in an interview:

*One HCP had a husband with the same problems as me, such as difficulties in handling all the issues with food. The HCP talked about these issues in a way and on a level without getting mad, because with a raised finger then you get really mad. (Interview participant 3)*

One HCP was particularly adept at modifying the program to be both comprehensive in scope and flexible in content to respond to spontaneous needs within the group. The HCP acknowledged and accepted issues important to the group by slowing down and paying deeper attention to crucial issues. The HCP demonstrated a purely empathetic focus by picking up and responding to verbal and nonverbal cues within the group. This approach succeeded partly because of a semi-structured format in which the HCP introduced a topic and followed the introduction with open-ended questions that elicited personal reflections from participants. The HCP focused more on connecting and guiding the group, which created a safe atmosphere in which the HCP succeeded in helping participants explore personal values. The following field note excerpt describes a meaningful and honest conversation about taboo issues:

*One participant told the group that her values were critical in terms of diabetes. “For me, it’s important to live a long and healthy life- so that I can be there for my family in the future and continue to be in nature.” “I’m extremely conscious of diabetes complications and terrified of getting my leg amputated. I know every single bit of dietary advice and know exactly how to choose low calorie muesli bars in the supermarket, but I just continue to eat it all until the whole packet is empty”. “I have too much time and eat between meals. Especially now, when I’m no longer in the labor market”. The rest of the group was wholeheartedly supportive and another participant expressed appreciation: “Your honesty and challenges really help the rest of the group”. (October 2016)*

In the interview, the participant described the experience of sharing personal issues:

*It was really difficult to be honest and tell about my frustrations. Now I’m really getting sad (tearfully). It’s really important and gives you something. But it’s very personal, because it’s your weaknesses that you are honest about. Normally I would not share such taboo issues. (Focus group participant 2)*

### Professional skills that were challenging

During the workshop, HCPs self-assessed professional skills within the Facilitator and Initiator roles to be the two most challenging roles. They linked challenges in facilitator skills to uncertainty about guiding the group back on track when the discussion took an unproductive or negative turn. Moreover, they found it fairly difficult to initiate motivation for behavior change. HCPs’ self-assessments corresponded with observations of the challenging professional skills during the educational programs and were confirmed in interviews.

#### Demanding to facilitate the process

HCPs seemed highly skilled in the Embracer role. However, many HCPs were unable to move from the Embracer who displays unconditional acceptance to the Facilitator who enables the process by having the courage to control, direct, and redirect the group in a timely way. As one HCP stated:

*“Everything we do as HCPs is often based on what the person thinks and feels. However, I feel a need to control or direct the group towards an overall aim”. (HCP interview 3 )*

It appeared that HCPs who were faced with unproductive conversations with participants were unable to change topics when needed. In one program, a program participant returned repeatedly to stories about old days in the army. In another program, the HCP had a long one-on-one conversation with a participant about a shift to a new general practitioner after the participant’s former GP retired. Consequently, engagement among the remaining group members completely drained away, creating the risk that their motivation to change would decrease. It seemed to be difficult for HCPs to keep the group on track and prevent unproductive drifts in discussions by either moving the group forward to a new focus or accelerating the conversation to a conclusion. This is described in a field note excerpt from observing an HCP facilitation exercise entitled “My Restaurant” [37]. Thirteen participants with T2DM attended:

*The HCP said: “Imagine you are in a restaurant...” The HCP gave instructions for the exercise while spreading pictures of different kind of meals out on the table. The participants were asked to work in small groups to choose one pictured meal. Subsequently, the HCP asked the groups to come up with suggestions on how a healthier restaurant meal could be planned. The first group had a picture of a meal based on a hamburger and suggested, “Skip the fries, bread with whole flour, less cheese, reduced-fat beef” etc. Meanwhile, several participants began to demonstrate resistance to the exercise because they wanted to allow occasional exceptions in their lives with diabetes. One participant glanced at another and whispered “Then I don’t bother going out”. Another confronted the HCP directly, saying “An infrequent restaurant visit shouldn’t be a guilty pleasure. For me, it’s the everyday life that counts”. The HCP addressed the resistance by saying, “Diabetes is demanding and doesn’t disappear if we continue to eat everything from a huge buffet because it will affect the blood sugar”. (April 2016)*

In this excerpt, the HCP kept the group on topic, even though it did not meet the current interest of the whole group. Moreover, participants became increasingly resistant as the process continued because they did not find the exercise relevant. It was difficult for the HCP to listen to the arguments against change without bias and then roll with resistance by accepting participants’ choices without approving the behavior. However, HCPs often chose to ignore, reject, or argue with resistant behavior. As one HCP stated in an interview about strategies for dealing with resistant behavior:

*“A participating husband was very good at—in a very ironic way—ignoring what we did and said. He asked several times, “Where is the cake? I want to go out and smoke! Why do we have to go out for a walk?” It was sort of very negative in a humorous way, you know? I simply ignored what he said”. (HCP interview 1)*

#### Difficult to initiate motivation

During observations, it became apparent that HCPs were challenged in the Initiator role. They were usually not responsive to participants ‘experiences, needs and concerns and did not incorporate them into the program. Frequently, HCPs allocated time at the beginning of the program to ask everyone in the group about their needs and expectations. However, no HCPs explored readiness to change and subsequently tailored the program based on readiness and needs. Participants simply articulated their expectations and needs, after which HCPs proceeded with their predetermined agenda, apparently expecting that individual needs would be fulfilled through the written curriculum. As one program participant described after participation in a program:

*“They tend to teach too much as if they read from a pamphlet, right. They have to deepen it one way or another”. (Focus group participant 12)*

Questions were most often closed-ended, which promoted short answers and little discussion. Open-ended questions were rarely used to encourage participants to reflect on important issues and guide them to explore reasons for change. The distinction between being facilitators rather than providers of information was often perceived by HCPs as too vague:

*I think that some of my patients will say, then I don’t get it all and my blind spots wouldn’t be disclosed. Then you stay where you are without the inspiration from outside (…) I’ll really need to tell them something more concrete. (HCP interview ﻿4)*

Occasionally, educational programs were characterized by engagement and collaborative learning techniques. This was evident when HCPs used the tool “My Eating Habits” [37]. The goal of the exercise was to reflect on and discuss food more broadly by incorporating psychosocial aspects of food. The exercise had 50 small cards containing statements from patients. The idea was to describe healthy and less healthy eating habits, including mental and practical aspects. Participants were asked to read and prioritize different quotations relevant to their relationships with food, with the goal of recognizing that some of the statements matched their experience. However, when HCPs used self-reflective tools to identify participants’ challenges and needs, they often ended the exercise after participants had identified their challenges. Moving from awareness of challenges towards acquiring new strategies and solutions was demanding and challenging for many HCPs. As one program participant described his perception of the exercise:

*Participant: “If the exercise gave me strategies to handle it [my eating habits], then it would have been meaningful”*

*Interviewer: “Do you think you got the tools to change your eating behavior?”*

*Participant: “No”. (Focus group participant 13)*

## Discussion

We explored the potential of a self-assessment tool based on The Health Education Juggler and techniques from MI in Groups to identify HCPs’ strengths and areas in need of professional development to aid effective facilitation of group-based, person-centered diabetes education**.** HCPs self-assessed professional skills within the Embracer and Translator roles as the ones they felt most skilled about and those within the Facilitator and Initiator roles as those that were most challenged. HCPs’ self-assessments corresponded with observations of their professional skills in practice. Thus, HCPs were able to self-assess their professional skills, which can serve as a starting point in planning of professional development program by organizing personalized professional development based on identified needs and challenges. To our knowledge, no studies have conceptualized the general components in self-assessing the comprehensive professional skills to facilitate high-quality group-based, person-centered diabetes education.

### The basis of the tool

The tool was based on MI in Groups and the Health Education Juggler as the two different models are complementary. The Health Education Juggler is an empirical, theoretical model describing ideal roles which makes it difficult to achieve in practice, whereas MI comprises a set of practical techniques to facilitate high-quality group-based, person-centered diabetes education. In MI, the decedent of Rogerian client-centered therapy [38], the roots in behavioral therapy, and the further drawing on a process-oriented view on group development [39] has shown to be efficacious in contributing to the field of facilitating health behavior change in groups [23]. Additionally, studies shows that the approach is highly applicable in facilitating group-based, person-centered diabetes education [23, 40]. MI in groups, has a particular focus on combining person-centered and goal-oriented strategies enabling HCPs to overcome the pitfalls of becoming either too directive or nondirective in their facilitation [23]. Nevertheless, MI has been criticized for being largely atheoretical [41]. Moreover, a weakness of using MI is the persuasive approach to direct the groups in dealing with ambivalence [42]. MI has proven effective in the field of alcohol treatment, although its less evident the approach today is widely applied into the context of health behavior change in chronic diseases [42]. Several person-centered models merely describe provider-patient communication in one-one consultations. The Health Education Juggler is to the best of our knowledge the first model to describe necessary roles to perform group-based patient education. However, a theoretical model can be difficult to apply in practice [10, 43].

### Why self-assess professional skills?

It has been argued that development of HCPs’ communication skills relies on knowledge of educational theories, critical reflection on professional skills, and participation in practice-oriented training programs [28]. However, studies have found that HCPs may perform patient education without reflecting on how they are performing it [28]. Some HCPs believe that communication skills are natural abilities, while others imply that professional skills rely on experience [44]. Thus, HCPs do not necessarily relate patient education to theories of teaching and training [44, 45]. Research indicates that professional skills can be primarily developed through critical self-reflection on skills [28]. Furthermore, evidence shows that lack of insight into personal professional skills is closely related to suboptimal professional performance [46]. Conscious efforts in self-reflection have been identified as essential in learning and developing professional skills with the ultimate goal of creating a mindful HCP capable of critical thinking [47]. Thus, self-assessments of skills in professional development programs have the potential to increase self-reflection, which is particularly beneficial when HCPs self-assessments and observations identify the same roles as challenging.

The question is whether HCPs are able to self-assess own professional skills. One study shows that HCPs who perform least well in external assessments tend to overrate their own performance [48]. Other studies have found that self-assessment of person-centered methods was essential for continuing education to promote professional growth, integration of theory into practice, and critical thinking [49]. In particular, self-assessment was found to have a greater impact on the process of self-reflection and was associated with a more positive or meaningful learning experience [50]. Moreover, studies have found that participation and engagement in general more likely promotes positive outcomes in the field of learning [51].

### Professional development- how to?

In general, the benefits of self-assessment for professional development are twofold. First, the tool could be useful in deepening HCPs’ theoretical understanding of how person-centeredness can be promoted in relation to the four Health Education Juggler roles. Second, self-assessment promotes self-reflection and awareness of the professional skills that the HCPs need to develop. However, a self-assessment tool for professional development cannot stand alone. Empowering HCPs to master group-based, person-centered skills may be a lengthy process [23]. Increasing skills requires knowledge in the theoretical paradigm, conscious self-reflection, and participation in practice-oriented training programs. Studies show that teaching communication skills is highly effective if they contain role-play or video-recordings of practice, followed by feedback and small group discussions, noting the importance of continuous practice to maintain skills over time [23, 52, 53]. It is clear that a self-assessment tool for professional development can be used as a first step to explore HCPs’ professional development needs.

### The complexity of juggling roles

The tool to self-assess professional skills is not suitable for exploring HCPs’ ability to juggle the four roles of the Health Education Juggler model because it only assesses skills within roles about which HCPs feel skilled and challenged. The health Education Juggler model refers to the importance of juggling all four roles. An appropriate method for developing and improving the ability to juggle between roles could be video recording followed by careful feedback from an experienced mentor, including questions that enhance self-reflection. Individual HCPs have different strengths and weaknesses in relation to the different roles. Thus, when forming a HCP team to promote group-based, person-centered diabetes programs, it would be valuable to strategically combine team members with different strengths and weaknesses to increase the team’s overall capability to successfully enact all four different Health Education Juggler roles. Doing so would likely increase each HCP’s skills and further increasing the quality of group-based, person-centered diabetes education.

### Implication for practice

The Health Education Juggler tool for professional development is a promising approach to self-assessing professional skills for facilitating group-based, person-centered diabetes education programs. In particular, the tool to self-assess professional skills is a learner-centered approach that supports self-reflection, which emphasizes autonomy and, in turn, can increase personal engagement [27]. However, after its use, further training and supervision is subsequently needed to support and develop person-centered professional skills.

A self-assessment tool for professional development cannot replace expert-designed coding scales used to measure professional communication skills. However, this study shows that HCP self-assessments are consistent with practice observations. Further research is needed to identify the ways in which self-assessments can complement expert-designed coding scales rating HCPs’ communication skills.

#### Strengths and limitations

Field work made it possible to enter ‘the black box’ [54] and observe professional skills from the inside. It also strengthens the study that the workshops included a multidisciplinary team of experienced HCPs recruited from the same educational program as observed. Additionally, an important strength of the study was that the knowledge obtained in the professional development workshops was relevant to the HCPs participating in the workshops—they were the primary consumers of the findings.

One limitation of the self-assess tool was that it assessed the most skilled and challenged Health Education roles which might be a too narrow categorization. The two polarities may not represent different entities as some HCPs may consider themselves skilled in one role while at the same time considering that particular role as most challenging. Another limitation was the impossibility of observing all HCPs that participated in the workshops. Nine HCPs from the workshop were observed in practice. However, all HCPs in the workshops were from the same educational program. Thus, it was not possible for all the observed HCPs to participate in the workshop due to organizational changes and some HCPs changed job.

## Conclusions

This study compared HCPs’ self-assessments of professional skills with the findings from interviews and observations of HCPs’ professional skills. We found that a tool to self-assess professional skills provided an effective way to promote self-reflections and identification of HCPs’ strengths and areas in need of professional development to aid effective facilitation of group-based, person-centered diabetes education. Their self-assessments corresponded to the interviews and observations of professional skills in practice and can form the basis for individualized professional development plans. Grounded in the Health Education Juggler and techniques from MI in Groups, the tool to self-assess professional skills can also promote self-reflections of the roles HCPs must juggle to facilitate group-based, person-centered diabetes education. Future research should examine the ways in which the self-assessment can augment or complement the current assessment standard of expert observations of HCPs and expert-designed coding scales rating professional communication skills.

## Additional file

Additional file 1: Appendix. Questionnaire, workshop 1 (questionnaire investigating HCPs’ professional background, level of experience, received supervision and training, and exploring readiness to change) (DOCX 67 kb)
