# Supplementary material for: The potential of a self-assessment tool to identify healthcare professionals’ strengths and areas in need of professional development to aid effective facilitation of group-based, person-centered diabetes education
Source: BMC Med Educ. 2017 Sep 18;17:166. doi: 10.1186/s12909-017-1003-3 (PMC5604418; doi:10.1186/s12909-017-1003-3)
Supplement: Additional file 1: Appendix. — Questionnaire, workshop 1 (questionnaire investigating HCPs’ professional background, level of experience, received supervision and training, and exploring readiness to change) (DOCX 67 kb) [file 12909_2017_1003_MOESM1_ESM.docx]

**Additional file 1**

**QUESTIONNAIRE, WORKSHOP 1**

1. **PERSONAL INFORMATION:**

(All responses will be kept confidential and will be used for research purposes only)

- 1. Name:
  2. Gender:
  3. Age:
  4. Your position:

1. **ARE YOU WORKING IN A MUNICIPALITY OR AT A HOSPITAL SETTING?**

- Municipality
- Hospital

1. **PLEASE MARK THE STATEMENT THAT BEST DESCRIEBES HOW EXPERIENCED YOU CURRENLY ARE IN FACILITATING GROUP-BASED, PERSON-CENTERED DIABETES EDUCATION PROGRAMS?**

- To a large degree
- To some degree
- Not much
- Not at all

1. **PLEASE MARK THE STATEMENT THAT BEST DESCRIEBES WHICH CONTINUING EDUCATION OR SUPERVISION WITHIN GROUP-BASED, PERSON-CENTERED METHODS YOU HAVE RECIEVED?**

- Postgraduate training

Please elaborate on type and level of education

- Courses

Please elaborate on type and level of education

- Supervision

Please elaborate on type and level of education

- Nothing

Please elaborate on type and level of education

1. **HOW OFTEN DO YOU USE METHODS TO FACILITATE GROUP-BASED, PERSON-CENTERED DIABETES EDUCATION PROGRAMS IN YOUR CURRENTLY WORK?**

- Regularly
- Occasionally
- Rarely
- Never

1. **PLEASE USE THE SCALE BELOW TO RATE HOW IMPORTANT IS IT FOR YOU TO USE GROUP-BASED, PERSON-CENTERED METHODS TO FACILITATE DIABETES SELF-MANAGEMENT EDUCATION PROGRAMS?**


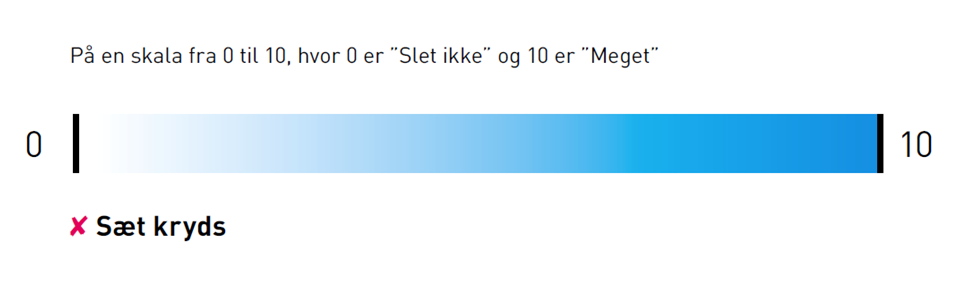


On a scale from 0 to 10 where 0 is ‘not at all’ and 10 is ‘very much’

On a scale from 0 to 10 where 0 is ‘nothing’ and 10 is ‘very much’

**Please mark**

On a scale from 0 to 10 where 0 is ‘nothing’ and 10 is ‘very much’

Please elaborate on your self-assessment:

1. **PLEASE USE THE SCALE BELOW TO RATE HOW INTERESSTED ARE YOU IN RECEIVING NEW INSPIRATION IN YOUR WORK WITH FACILITATING GROUP-BASED, PERSON-CENTERED DIABETES EDUCATION PROGRAMS?**


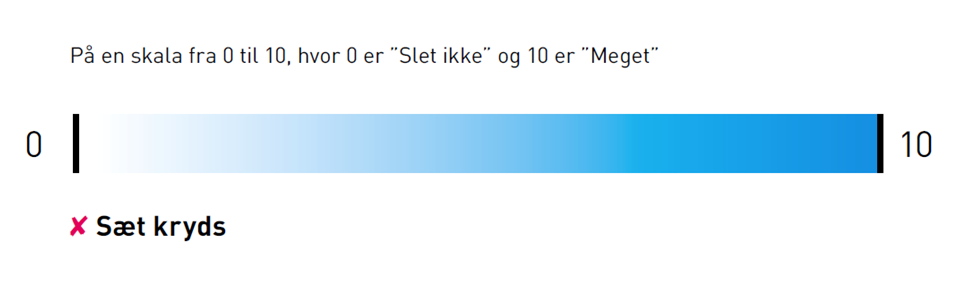


On a scale from 0 to 10 where 0 is ‘not at all’ and 10 is ‘very much’

**Please mark**

Please elaborate on your self-assessment:
